# Immune cell expression of TGFβ1 in cancer with lymphoid stroma: dendritic cell and regulatory T cell contact

**DOI:** 10.1007/s00428-018-2336-y

**Published:** 2018-03-28

**Authors:** Haruo Ohtani, Toru Terashima, Eiichi Sato

**Affiliations:** 1 Department of Pathology, Mito Saiseikai General Hospital, 3-3-10 Futabadai, Mito, Ibaraki, 311-4198 Japan; 2 Department of Pathology, National Hospital Organization, Mito Medical Center, Ibaraki, Japan; 3 Department of Surgery, National Hospital Organization, Mito Medical Center, Ibaraki, Japan; 4 Department of Pathology (Medical Research Center), Institute of Medical Science, Tokyo Medical University, Shinjuku-ku, Tokyo, Japan

**Keywords:** TGFβ, Lymphocyte-rich gastric cancer, Dendritic cell, Regulatory T cell, Tertiary lymphoid tissue

## Abstract

Although cancer tissue generally shows limited immune responses, some cancers abound with lymphocytes, which generally show favorable prognosis. These cancers, despite their rarity, are important in analyzing immune responses in cancer tissue. Transforming growth factor β1 (TFGβ1) is a multifunctional cytokine, generally having an immunosuppressive function. The present study analyzes the in situ TGFβ1 expression in 23 cases of lymphocyte-rich gastric carcinomas (Ly-rich GCs) using immunohistochemistry and in situ hybridization. Immunohistochemistry revealed that latency-associated peptide (LAP) of TGFβ1 was localized in mainly immune cells in all cases, which was more abundant than in control GCs. Expression of LAP by cancer cells was only focal. In situ hybridization also confirmed abundant TGFβ1 mRNA expression in the lymphoid stroma. Double immunofluorescent microscopy identified LAP^+^ cells as macrophages, dendritic cells, and part of T cells. Close cell-to-cell contact was observed between LAP^+^ dendritic-shaped cells and FoxP3^+^ regulatory T cells (T_reg_ cells). Mature dendritic cells in Ly-rich GCs expressed LAP more frequently than those in the secondary lymphoid organs. Our data revealed abundant expression of TGFβ1 in immune cells with contact to T_reg_ cells in lymphoid stroma, which is consistent with the notion that TGFβ1 is one of the immunosuppressive factors in cancer stroma.

**Electronic supplementary material:**

The online version of this article (10.1007/s00428-018-2336-y) contains supplementary material, which is available to authorized users.

## Introduction

Transforming growth factor β (TGFβ) is a multifunctional cytokine, with recent emphasis on its immunoregulatory function [1]. In cancer, TGFβ could both promote and suppress tumor growth [2, 3]. Its immunosuppressive function has drawn much attention due to recent progress in cancer immunotherapy [4]. TGFβ, generally believed to be produced by cancer cells, could suppress the function of tumor-infiltration of both adaptive and innate immune cells (including CD4^+^ or CD8^+^ T cells, dendritic cells, natural killer cells, neutrophils, and macrophages), and thus cancer tissues are generally under an immunosuppressive microenvironment [5–7].

The present authors have pathologically analyzed human cancer with prominent lymphocytic infiltrate, including gastric [8, 9] and breast cancers [10]. Such cancers, associated with an abundance of immune cells, generally show a favorable prognosis (see Supporting Information 1 for histological details). However, occurrence of cancer tissue itself demonstrates that such cancers at the same time exert vigorous immunosuppressive mechanisms to dampen possible immune cell attacks. Infiltration of cancer tissue by regulatory T cells (T_reg_ cells) is one such mechanism, with their presence related to poor survival of patients [11, 12]. We analyzed T_reg_ cells confirming that T_reg_ cells positively correlated with immune effector cells [13].

The present study is designed to analyze the in situ localization of TGFβ1 in cancers with prominent lymphocytic infiltrate. As a representative example, here we used lymphocyte-rich gastric cancers (Ly-rich GCs) as a continuation of our study [8, 9, 13]. We used two sets of control: (1) control/conventional gastric cancers (GCs) without well-formed lymphoid stroma and (2) the secondary lymphoid organs (lymph nodes, Peyer patches, or tonsils). The theoretical basis of the second set of control is that immune responses in cancer tissue may simulate the structure of these secondary lymphoid organs (i.e., tertiary lymphoid tissue) when such immune responses are vigorous [9]. Herein, we reveal (a) immune cell predominant expression of TGFβ1, (b) the identification of TGFβ^+^ immune cell types, and (c) close cell-to-cell contact between TGFβ1^+^ dendritic-shaped cells and T_reg_ cells. Because previous papers on the tissue distribution of TGFβ did not deal with immune responses in gastric cancer tissue [14–16], the present paper describes detailed TGFβ1 localization in immune cells in human cancer tissues for the first time.

## Materials and methods

### Materials

The present study is a retrospective study using archival materials in the Department of Pathology, mainly Mito Medical Center and partly Mito Saiseikai General Hospital. Ly-rich GCs in this paper include typical lymphoepithelioma-like carcinoma (LELC) (or gastric cancer with the lymphoid stroma), which were characterized by poorly differentiated, solid-type cancer cells surrounded by abundant tumor-infiltrating lymphocytes (TILs), and LELC-like carcinoma showing any type of cancer with TILs in the whole stroma (for details, see Fig. 5 in Appendix 1). We used 23 cases of surgically removed Ly-rich GCs (Epstein-Barr virus [EBV]^+^, 14 cases; EBV^−^, 9 cases) (median age 65 years, range 47–84, M/F ratio = 16/7) and consecutively sampled 35 lesions of control (i.e., conventional) GCs (all EBV^−^) in 32 patients (median age 73 years, range 48–87, M/F ratio = 25/7). The method for the EBV detection was described previously [9]. Of nine cases of EBV^−^ Ly-rich GCs, three were considered to be in the microsatellite instability status (Fig. 6 in Appendix 1). Control GCs were associated with either TIL responses along the invasive margin or a general paucity of TILs. Intramucosal carcinomas (pTis or pT1[M]) were not included in this study because typical lymphocyte-rich stroma was observed only when carcinoma cells invade the submucosa. The stage of cancer was classified as described by Tumor-Node-Metastasis (TNM) classification (7th ed.) [17]. The stage, histological typing, and follow-up analysis are shown in Tables 1 and 2 and Fig. 7 in Appendix 1. For the control of dendritic cells, 25 lesions of surgically resected secondary lymphoid organs from 22 patients were used (8 lymph nodes, 7 tonsils, 4 spleens, 4 appendices, 2 Peyer’s patches) (median age 28, range 5–67, M/F ratio = 10/12). The original diagnosis included tonsillitis, abdominal trauma, and gastrointestinal cancer.

### Immunohistochemistry

All histochemical data were obtained using formalin-fixed and paraffin-embedded tissue sections. The primary antibodies used in this study were antigen affinity-purified goat polyclonal antibody to human LAP (TGFβ1) (R&D systems, Minneapolis, MN; no. AF-246-NA; used at 1:250 = 0.4 mg/mL), mouse monoclonal antibodies to human CXCR3 (CD183) (clone 1C6, IgG1; BD, Franklin Lakes, NJ; used at 1:400 = 1.25 μg/mL) and to forkhead box P3 (FoxP3) (clone 236A/E7, IgG1; Abcam, Cambridge, MA; used at 1:100), and antigen affinity-purified rabbit polyclonal antibody to human Smad3C (Immuno-Biological Laboratories Co. [IBL], Fujioka, Japan; no. 28031, used at 1:125 = 0.4 μg/mL). For negative controls, the primary antibodies were replaced by either normal goat- or rabbit IgG (IBL). The incubation time of the primary antibodies was overnight. The immunohistochemical methods were described previously [9]. In brief, heat antigen retrieval was performed in high pH buffer (S3308, DAKO) at 95 °C for 60 min. The secondary antibodies included horseradish-peroxidase conjugated anti-goat simple stain (Nichirei, Tokyo, Japan), and anti-mouse or anti-rabbit envisions (DAKO). Diaminobenzidine (DAB) (DAKO) was used as the chromogen. Endogenous peroxidase activity was inactivated by immersing tissue sections in 3% H_2_O_2_ for 5 min after incubation with primary antibodies.

### Cell counting

The distribution density of LAP^+^ immune cells, CXCR3^+^ cells, and FoxP3^+^ cells were manually counted as follows: total positively stained cells were counted using an ocular grid (10 × 10 mm lattice) with a × 400 microscopic field (a × 40 objective lens and × 10 ocular lens using a BX51 microscope, Olympus, Tokyo, Japan). The area of one lattice was 0.0625 mm^2^. At least three areas were counted in each case, and the numbers were averaged. In this analysis, the most densely distributed areas were selected. All statistical analyses were performed using IBM SPSS statistics software, version 21 (IBM Inc., Armonk, NY, USA).

### In situ hybridization for TGFβ1 mRNA

TGFβ1 mRNA was detected by RNAscope 2.5 HD Reagent Kit (Advanced Cell Diagnostics, Hayward, CA) according to the manufacturer’s instructions. As a minor modification, an OPAL 520 TSA detection system (PerkinElmer, Waltham, MA) was used for fluorescent labeling instead of chromogenic coloring. As a negative control, a bacterial *dapb* gene was employed.

### Double-labeling immunofluorescence method for LAP and CD83, LAP and CD68, LAP and FoxP3, and LAP and CD3

Formalin-fixed and paraffin-embedded tissue sections were used. Antigen retrieval was performed as described above. The sections were incubated with a mixture of goat anti-human LAP (1:75 = 1.25 μg/mL) and mouse monoclonal anti-human CD83 (1:8; clone 1H4b, Novocastra-Leica Microsystems, Benton Lane, UK), anti-CD68 (1:80; clone PG-M1, DAKO) or anti-CD3 (1:8; clone F7.2.38, DAKO) overnight. Alexa Fluor 488-labeled donkey anti-goat IgG (1:100 = 20 μg/mL, Molecular Probe, Carlsbad, CA) and Alexa Fluor 555-labeled donkey anti-mouse IgG (1:100 = 20 μg/mL) were applied in a mixture for 30 min. After DAPI (Molecular Probe) nuclear staining, specimens were mounted with ProLong Gold (Molecular Probe). Immunofluorescent observation was performed with a confocal laser scanning microscope (TCS SP5, Leica Microsystems, Wetzlar, Germany) or with a Nikon E800 microscope (Nikon, Tokyo, Japan). For negative control, the primary antibodies were replaced by either non-immunized goat IgG (IBL; 1.25 μg/mL) or control mouse IgG1 (DAKO; 1:100 = 4 μg/mL).

### Double-labeling chromogenic immunohistochemistry for CD68-LAP, CD83-LAP, and FoxP3-LAP

The immunoperoxidase method for CD68, CD83, and DC-sign was performed as described for single immunohistochemistry. Tissue sections were then re-treated with Tris-EDTA antigen retrieval solution at 95 °C for 20 min to inactivate antibodies and enzymes used in the first step. Then, immunohistochemistry for LAP was performed. The combination of chromogens used was as follows: DAB (brown; DAKO), Vector SG (dark blue/gray; Vector Laboratories, Burlingame, CA) and Vulcan Fast Red (red; Biocare, Concord, CA), DAB (brown; DAKO). For Vulcan Fast Red, we used anti-mouse simple stain conjugated with alkaline phosphatase (Nichirei).

## Results

### TGFβ1 expression by mainly immune cells in Ly-rich GCs

**Fig. 1:**
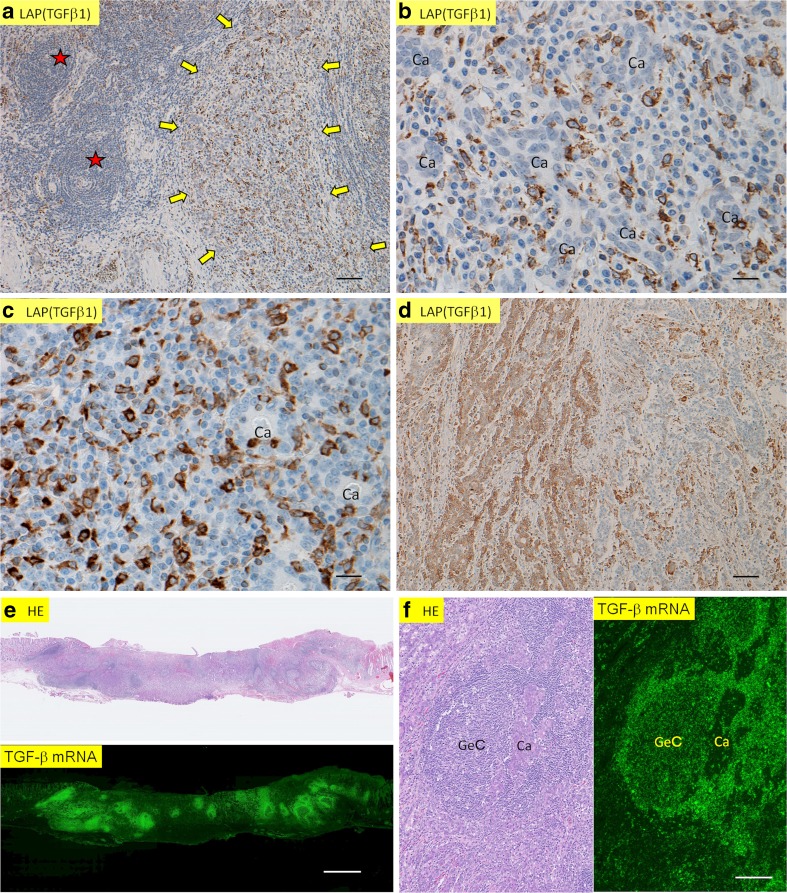
In situ localization of TGFβ1 in Ly-rich GCs. **a** Immunohistochemistry shows that LAP (TGFβ1) (brown) is expressed in immune cells (indicated by arrows). Asterisks indicate lymphoid follicles, where positive cells were sparse. **b** A higher magnification of Fig. 1a shows that positive cells are oval, dendritic, or round in shape. Carcinoma cells (Ca) are negative for LAP (TGFβ1). **c** Another example of immune cell expression of LAP (TGFβ1) in lymphocyte-rich stroma. **d** In this case of Ly-rich GC, carcinoma cells are immunolabeled for LAP (TGFβ1) in the left half, while the right half shows negative staining for LAP in cancer cells (i.e., the presence of heterogeneity). **e** A pair of HE (upper) and in situ hybridization for TGFβ1 (lower) in Ly-rich GC. Signals are expressed by green light. Note abundant signals in the areas of lymphoid stroma. **f** A pair of higher magnification in Fig. 1e. HE (left) and in situ hybridization (right). Note that TGFβ1 mRNA is mainly expressed in lymphoid stroma except for germinal center (GeC). Cancer cells (CA) do not express clear signals for TGFβ1. Scale bars, 20 μm (b, c), 100 μm (a, d, f), and 1 mm (e)

In this paper, we mainly dealt with stromal immune cells, because intraepithelial lymphocytes are difficult to identify in Ly-rich GCs suing immunohistochemical specimens. Immunoreactivity for latency-associated peptide of TGFβ1 (LAP [TGFβ1]) [18] was abundantly observed among immune cells in the lymphoid stroma in all 23 cases of Ly-rich GCs (Fig. 1a–c), irrespectively of EBV status. The immunoreactive cells were mononuclear, usually dendritic/reticular and partly small-round in shape (Fig. 1b, c). For negative control, the anti-LAP (TGFβ1) antibody was replaced by non-immunized goat IgG, resulting in no reactivity (Fig. 8-1 in Appendix 2). By contrast, cancer cells showed various degrees of immunoreactivities in only 3 of 23 cases (Fig. 1d). The three cases were 1 EBV^*+*^ case in which approximately 10% of cancer cells were positive for LAP (TGFβ1) and 2 EBV^−^ cases in which approximately 50 and 20% of cancer cells expressed LAP (TGFβ1).

To check the reliability of the above-mentioned results, we analyzed TGFβ1 mRNA expression using ISH in four representative Ly-rich GCs (all EBV^+^). At panoramic view, immunofluorescent signals were observed in areas exactly corresponding to the lymphoid stroma (Fig. 1e). At higher magnification, areas of intramucosal carcinoma without a lymphoid stroma showed inconspicuous signals for TGFβ1, contrasted by prominent signals in the lymphoid stroma (Fig. 1f). The results at this point clearly showed that TGFβ1 is produced and expressed by mainly immune cells in Ly-rich GCs.

**Fig. 2:**
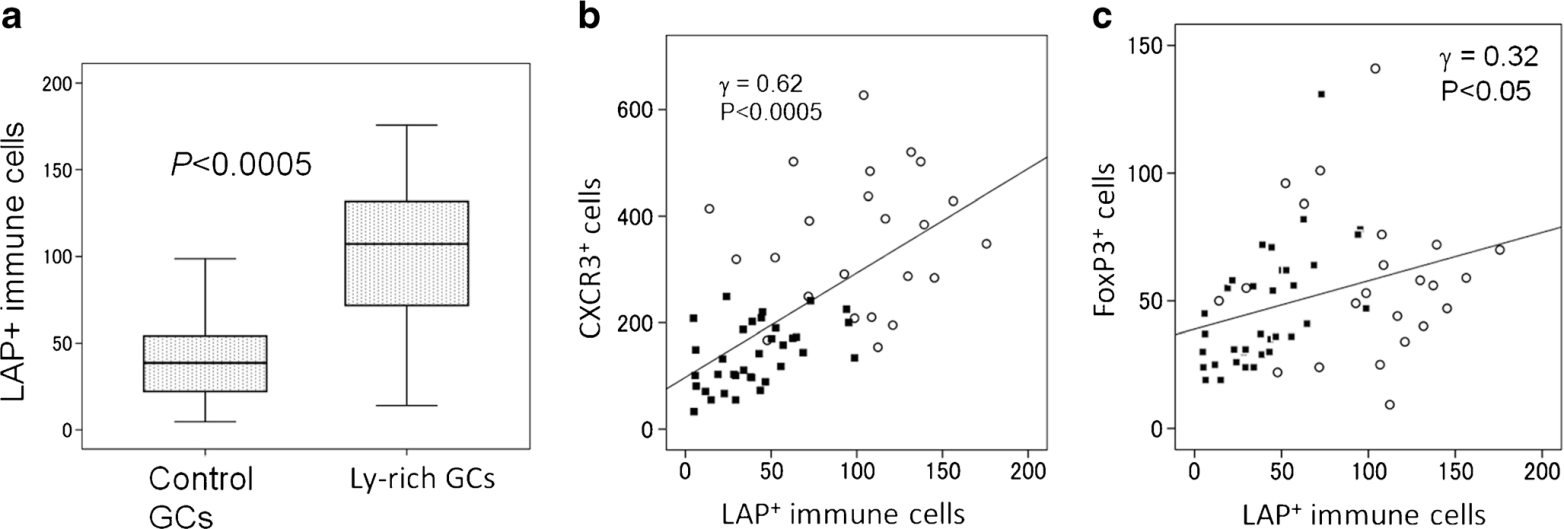
Quantification results of LAP (TGFβ1)^+^ immune cells. In control GCs, areas with the densest distribution were analyzed. **a** The number of LAP (TGFβ1)^+^ immune cells are larger in Ly-rich GCs than in control GCs. Box-whisker plots. Vertical line, the number of cells in one unit area (one lattice in a × 400 field, 0.0625 mm^2^). *P* values tested by Mann-Whitney *U* test. **b**, **c** Results of correlation analyses. The number of LAP (TGFβ1)^+^ immune cells correlated strongly with that of CXCR3^+^ cells (b), and weakly with FoxP3^+^ T_reg_ cells (c) in all GCs. Open circles, Ly-rich GCs. Black boxes, control GCs. Vertical line, the number of immunoreactive immune cells in one unit area (one lattice in a × 400 field, 0.0625 mm^2^). Statistical analysis by Spearman’s test

Thirty-five cases of control (or conventional) GCs without abundance of lymphoid stroma (all EBV^−^) showed immunoreactivity for LAP (TGFβ1) in immune cells where lymphocytic infiltrate was observed. This was particularly noted along the tumor-host interface (invasive margin) (Fig. 8-2 in Appendix 2, left). Tumor cells expressed LAP (TGFβ1) in 10 of 35 cases of control GCs (Fig. 8-2 in Appendix 2, right), of which 5 cases showed positivity in more than 10% of carcinoma cells, and the other 5 cases showed positivity in less than 10% of carcinoma cells.

### Quantitative analysis of LAP (TGFβ)^+^ immune cells in cancer tissues

To check the significance of LAP (TGFβ1)^+^ immune cells, we quantified them in all cancer cases. Its distribution density was more abundant in Ly-rich GCs than in control GCs (Fig. 2a). (Note that areas with lymphocytic infiltrate were selectively measured in control GCs.) Next, we compared LAP expression with other cell types including CXCR3^*+*^ and FoxP3^*+*^ cells, which represent type 1 immune cells (cytotoxic T cells and T helper type 1 cells) and T_reg_ cells [9]. The distribution density of LAP (TGFβ1)^+^ immune cells in total cases strongly correlated with that of CXCR3 and weakly with that of FoxP3 (Fig. 2b, c). This indicates that the expression of TGFβ1 in immune cells correlated with the degrees of immune cell infiltrate in cancer tissue. This suggests a quantitative difference between Ly-rich- and control GCs.

### Identification of LAP(TGFβ)^+^ immune cells as macrophages, dendritic cells, and T cells

**Fig. 3:**
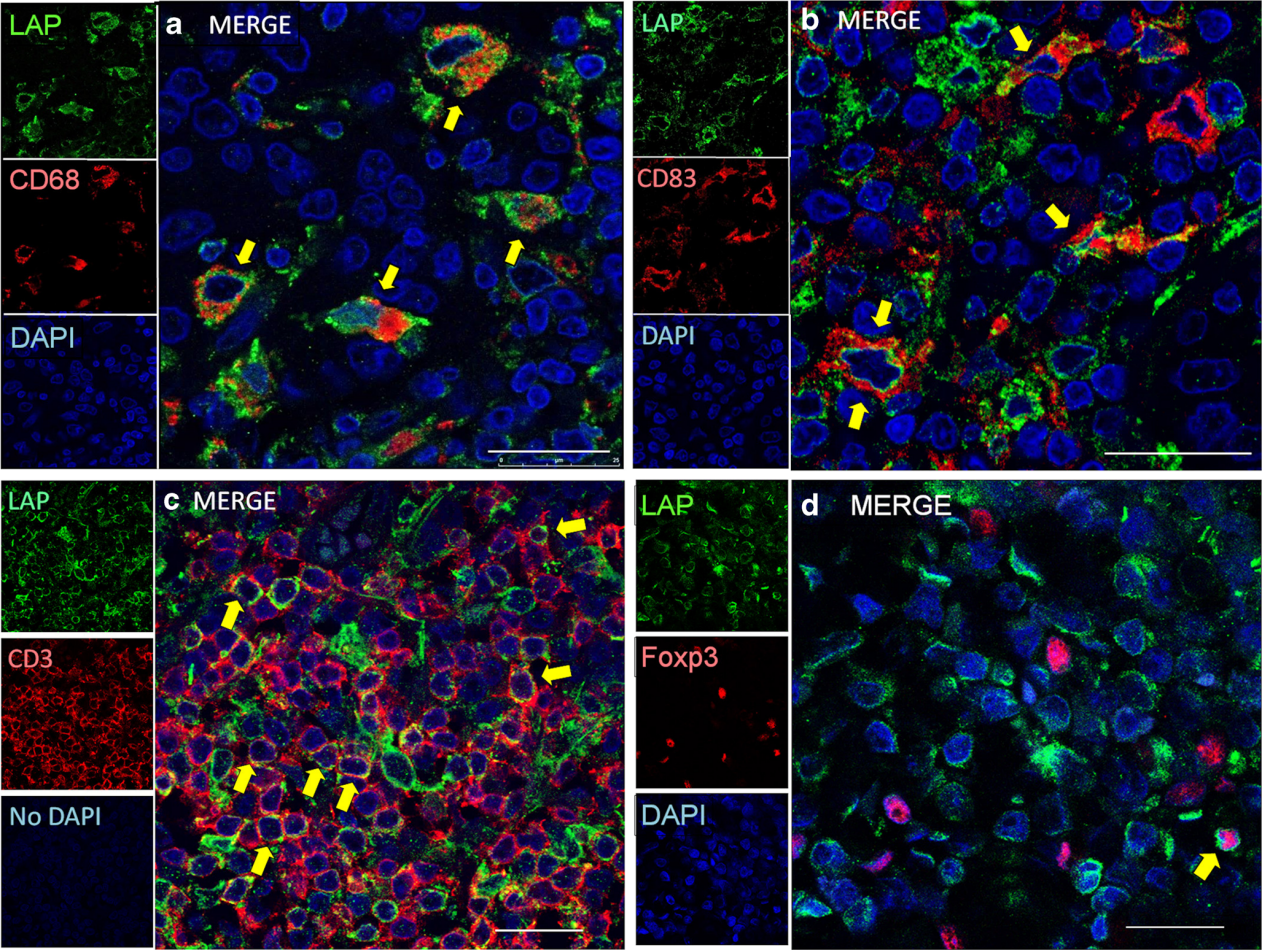
Double immunofluorescence for LAP (TGFβ1) (green) plus CD68 (red)(**a**), CD83 (red)(**b**), CD3 (red)(**c**), and FoxP3 (red)(**d**) by confocal laser scanning microscopy. Arrows, double positive cells. LAP (TGFb1)^+^ immune cells include macrophages (a) (most abundant), mature conventional dendritic cells (cDCs) (b), and a part of T cells (c). It is noteworthy that T_reg_ cells (d) infrequently co-express LAP (TGFβ1). Scale bars, 10 μm (a–d)

**Fig. 4:**
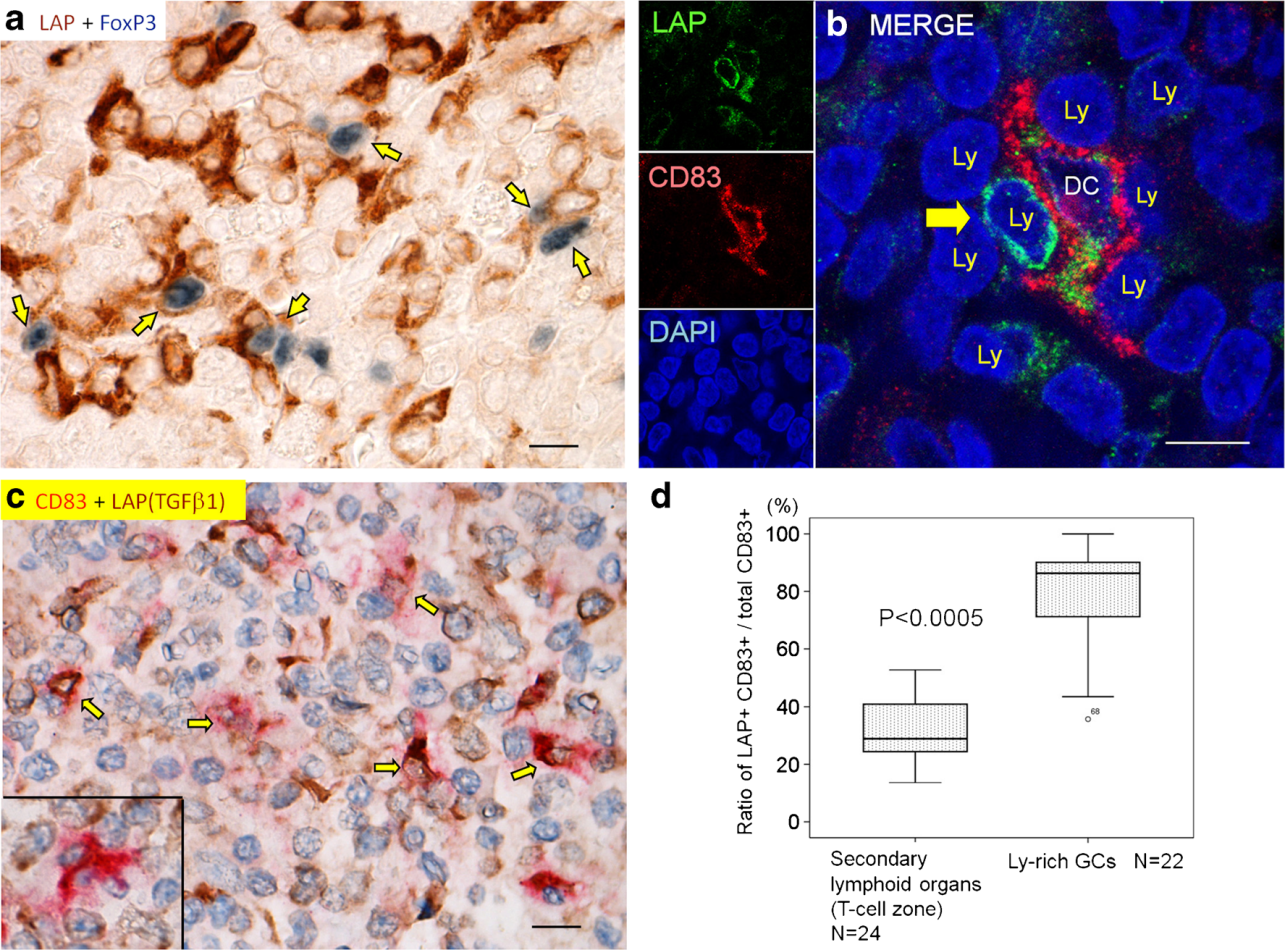
Immunohistochemical analyses in Ly-rich GCs. **a** LAP (TGFβ1)^+^ cells (blue) harbor FoxP3^+^ T_reg_ cells (dark blue) in a cell-to-cell contact (double chromogenic immunostaining). **b** Close cell-to-cell contact between LAP (TGFβ1)^+^ (green), CD83^+^ (red) mature cDC, and LAP (TGFβ1)^+^ lymphocyte (Ly) by confocal laser scanning microscopy. **c** Double chromogenic immunohisochemistry for LAP (TGFβ1) (brown) + CD83 (red). Double positive cells are expressed by arrows. Inset shows a mature cDC not labeled for LAP (TGFβ1). Quantification in Fig. 4d was done with this method. Scale bars, 10 μm (a–c). **d** Quantification analysis shows a higher ratio of LAP (TGFβ1) expression in CD83^+^ mature cDCs in Ly-rich GCs than in the T cell zone of the secondary lymphoid organs. Box-whisker plots. *P* values by Mann-Whitney *U* test. In Ly-rich GCs, areas with lymphoid follicles were excluded

LAP (TGFβ1)^+^ immune cells were identified as macrophages, classical/conventional dendritic cells (cDCs) (including both immature and mature cDCs), and part of T cells including T_reg_ cells in Ly-rich GCs. Because LAP (TGFβ1)^+^ cells co-expressed CD68 (macrophages or immature cDCs), CD83 (mature cDCs), CD209 (DC-SIGN) (immature DCs), CD3 (T cells) or FoxP3 (T_reg_ cells) (Fig. 3a~d; Fig. 8-3 in Appendix 2). Among LAP^+^ immune cells, CD68^+^ cells were most frequent. It was noteworthy that FoxP3^+^ T_reg_ cells were only focally double-positive for LAP (TGFβ1) (Fig. 3d; Fig. 8-4 in Appendix 2).

We next analyzed the secondary lymphoid organs by the same method to confirm that the same cell types expressed LAP (TGFβ1) in the T-zone (paracortex) (Figs. 8-5 and 8-6 in Appendix 2). It is noteworthy that macrophages in the germinal center were negative for LAP (TGFβ1). The results here indicate a qualitative similarity between Ly-rich GCs and the T cell zone of secondary lymphoid organs from a viewpoint of TGFβ expression in immune cells.

### Cell-to-cell contact between LAP^+^ dendritic-shaped cells and lymphocytes in Ly-rich GCs

Inducible T_reg_ cells are induced in peripheral tissues under stimuli of TGFβ and IL-2, with DCs being crucial in this process [19]. We searched for in situ cellular relationship between LAP (TGFβ1)^+^ cells and FoxP3^+^ T_reg_ cells. LAP (TGFβ1)^*+*^ dendritic/reticular-shaped cells (DCs or DC-like cells) were frequently in close contact with lymphocytes including FoxP3^+^ cells in Ly-rich GCs (confirmed in six representative cases) (Fig. 4a; Fig. 8-7 in Appendix 2).

Next, we analyzed more detailed cell-to-cell interactions. In three representative Ly-rich GCs, LAP (TGFβ1)^+^ CD83^+^ mature cDCs extended their cytoplasmic processes and harbored several lymphocytes, including LAP (TGFβ1)^+^ lymphocytes (Fig. 4b, indicated by an arrow). This close cell-to-cell contact to lymphocytes is typical for cDCs.

We then analyzed the in situ expression of Smad3C (phosphorylated Smad3) because it is an essential intracellular signal transducer of TGFβ, and Smad3 is important for immunity [20]. Smad3C was ubiquitously expressed by both carcinoma cells (nearly all) and lymphocytes (approximately 50% to nearly 100%) (Fig. 8-8 in Appendix 2), which was confirmed in 12 cases of Ly-rich GCs. This suggests that lymphocytes in cancer stroma have a potentiality to receive TGFβ signaling.

### Higher LAP positivity in mature cDCs in Ly-rich GCs than in the secondary lymphoid organs

The previous sections showed that Ly-rich GCs contained LAP^+^ CD83^+^ mature cDCs. We explored whether this is specific to cancer or if it is ubiquitous. The stroma of Ly-rich GCs can be considered as “tertiary lymphoid tissue” [9]. Therefore, we used 21 cases of secondary lymphoid organs as a control to explore how the cancer stroma differs from the secondary lymphoid organs. By chromogenic immunohistochemistry, double positive cells for LAP (TGFβ1) and CD83 are expressed by co-localization of red and brown colors (Fig. 4c). In the secondary lymphoid organs, the positivity rate of LAP among cDCs (CD83^+^ LAP^+^ cells/total CD83^+^ cells) were higher in the T cell zone of gut-associated organs (Peyer patch, mesenteric lymph nodes [MLNs], and appendix vermiformis) than in the T cell zone of other organs (tonsils and spleen) (Fig. 9a in Appendix 2). As shown in Fig. 4d, the positivity rate of LAP (TGFβ1) among cDCs was significantly higher in Ly-rich GCs than in the T cell zone of secondary lymphoid organs as a total (note that the areas of lymphoid follicles were excluded in Ly-rich GCs). The same result was obtained when we confined the secondary lymphoid organs to the gut-associated organs (Fig. 9b in Appendix 2). The total number of cDCs was higher in the T cell zone of secondary lymphoid organs than in Ly-rich GCs (Fig. 9c in Appendix 2). The data so far suggests a more immunosuppressive microenvironment in Ly-rich GCs than in the secondary lymphoid organs in respect to LAP (TGFβ1) expression in cDCs. In control GCs, CD83^+^ cells were generally sparse, and therefore this ratio could not be analyzed.

Next, we performed correlation analyses. In Ly-rich GCs, the number of total cDCs and that of LAP (TGFβ1)^+^ cDCs positively correlated with that of CXCR3^+^ cells, but did not correlate with that of FoxP3^+^ T_reg_ cells (Fig. 10a~c in Appendix 2).

## Discussion

The present histopathological study analyzed the in situ expression of TGFβ1 in human Ly-rich GCs to show that its expression is observed mainly in immune cells, but only focally in cancer cells. The number of TGFβ1^+^ immune cells correlated with those of CXCR3^+^ cells and T_reg_ cells, which demonstrates more immune-cell responses in Ly-rich GCs than in control GCs. Double staining confirmed TFGβ1 expression in macrophages and/or immature cDCs and mature cDCs, and some T cells. TGFβ1^+^ dendritic cells harbor lymphocytes including T_reg_ cells in their cytoplasmic processes.

T_reg_ cells are one of the important immunosuppressive cells. The expression and production of TGFβ in macrophages or immature cDCs is well known, and such cells could induce T_reg_ cells [21]. In fact, we have shown here that TGFβ1-expressing and dendritic-shaped cells harbor lymphocytes including T_reg_ cells along their cytoplasmic processes. These are consistent with observations that T_reg_ cells are induced in the peripheral tissue from naïve CD4^+^ T cells by TGFβ expressed on cDCs. Such T_reg_ cells also express TGFβ in a mouse model [19]. In addition, human DCs activated by cancer cells or tumor-associated antigens can induce T_reg_ cells by producing TGFβ [22, 23]. Therefore, our morphological data are consistent with close relationship between T_reg_ cells and LAP (TGFβ1)^+^ cDCs. We have shown here that the number of LAP (TGFβ1)^+^ immune cells (as a total) correlated with that of T_reg_ cells, but that of LAP (TGFβ1)^+^ cDCs did not. These data would suggest that not a minor part of T_reg_ cells infiltrates lymphoid stroma probably independently of LAP (TGFβ1) ^+^ cDCs, and T_reg_ cells may also be induced in cancer stroma. LAP (TGFβ1)^+^ immune cells also include significant number of CD68^+^ macrophages. Therefore, relationship between LAP (TGFβ1)^+^ CD68^+^ cells and T_reg_ cells is to be analyzed in future studies. Taken together, our data suggest that TGFβ1 could be one of the candidates of immunosuppressive factors in cancers, and that TGFβ1 has a potential to promote cancer growth together with T_reg_ cells. Functional analyses would be required in future studies.

The distinction between macrophages and DCs is difficult [24], particularly in inflammatory lesions in the peripheral organs including Ly-rich GCs. Therefore, future multi-color immunohistochemistry will be required for more-detailed in situ characterization of TGFβ1^+^ dendritic-shaped cells in cancer tissues.

Next, we need to discuss the differences between the present study and our previous study where we observed TGFβ1 expression in stromal fibroblasts and macrophages in scirrhous gastric carcinoma [14]. Formerly, we observed TGFβ1 expression in endoplasmic reticulum in spindle-shaped macrophages. Therefore, it is reasonable to speculate that TFGβ1^+^ spindle-shaped “fibroblasts” in scirrhous carcinoma in our previous study were in fact spindle-shaped macrophages. Cancer cell expression of TGFβ1 in the previous study is consistent with the present study on control GCs.

We have already observed that the lymphoid stroma in Ly-rich GCs is similar to lymphoid tissue, postulating that the lymphoid stroma corresponds to the tertiary lymphoid tissue [9]. This concept was later analyzed in details [25]. Our data here indicate that the lymphoid stroma of cancer are considered to be under more immunosuppressive microenvironment than the secondary lymphoid organs as shown by higher TGFβ expression rate in cDCs. Not only Ly-rich GCs but also control GCs showed a similar distribution of TGFβ1 in the areas with lymphocyte-present stroma, particularly along the invasive margin. This suggests that our data could be widely applicable to various cancers not associated with lymphoid stroma. In conclusion, we would be able to judge the following: (a) TGFβ1 is mainly expressed in immune cells (including macrophages and cDCs) with a close contact to T_reg_ cells in lymphoid stroma, (b) Ly-rich GCs quantitatively differ from control GCs from the viewpoint of TGFβ expression in immune cells, and (c) the lymphoid stroma of Ly-rich GCs is quantitatively different from the T cell zone of secondary lymphoid organs from the viewpoint of TGFβ expression rate in cDCs. Finally, we need to note that TGFβ could be a target of cancer immunotherapy in combination with, for example, immune checkpoint blockage therapy [26, 27]. Our study could be a basis of such therapies.

## Electronic supplementary material

Appendix 1 Supplementary data in the Materials and Methods section. (DOCX 788 kb)

Appendix 2 Supplementary data in the Results section. (DOCX 4299 kb)

## References

[1] Chen W, Ten Dijke P (2016). Immunoregulation by members of the TGFβ superfamily. Nat Rev Immunol.

[2] Bierie B, Moses H (2006). TGFβ: the molecular Jekyll and Hyde of cancer. Nat Rev Cancer.

[3] Tian M, Schiemann WP (2009). The TGF-beta paradox in human cancer: an update. Future Oncol.

[4] Gigante M, Gesualdo L, Ranieri E (2012). TGF-beta: a master switch in tumor immunity. Curr Pharm Des.

[5] Flavell RA, Sanjabi S, Wrzesinski SH, Licona-Limón P (2010). The polarization of immune cells in the tumour environment by TGFβ. Nat Rev Immunol.

[6] Tu E, Chia PZ, Chen W (2014). TGFβ in T cell biology and tumor immunity: angel or devil?. Cytokine Growth Factor Rev.

[7] Speiser DE, Ho PC, Verdeil G (2016). Regulatory circuits of T cell function in cancer. Nat Rev Immunol.

[8] Saiki Y, Ohtani H, Naito Y, Miyazawa M, Nagura H (1996). Immunophenotypical characterization of Epstein-Barr virus-associated gastric carcinoma: massive infiltration by proliferating CD8^+^ T-lymphocytes. Lab Investig.

[9] Ohtani H, Jin Z, Takegawa S, Nakayama T, Yoshie O (2009). Abundant expression of CXCL9 (MIG) by stromal cells that include dendritic cells and accumulation of CXCR3^+^ T cells in lymphocyte-rich gastric carcinoma. J Pathol.

[10] Ohtani H, Mori-Shiraishi K, Nakajima M, Ueki H (2015). Defining lymphocyte-predominant breast cancer by the proportion of lymphocyte-rich stroma and its significance in routine histopathological diagnosis. Pathol Int.

[11] Tanchot C, Terme M, Pere H, Tran T, Benhamouda N, Strioga M, Banissi C, Galluzzi L, Kroemer G, Tartour E (2013). Tumor-infiltrating regulatory T cells: phenotype, role, mechanism of expansion in situ and clinical significance. Cancer Microenviron.

[12] Saito T, Nishikawa H, Wada H, Nagano Y, Sugiyama D, Atarashi K, Maeda Y, Hamaguchi M, Ohkura N, Sato E, Nagase H, Nishimura J, Yamamoto H, Takiguchi S, Tanoue T, Suda W, Morita H, Hattori M, Honda K, Mori M, Doki Y, Sakaguchi S (2016). Two FOXP3(+)CD4(+) T cell subpopulations distinctly control the prognosis of colorectal cancers. Nat Med.

[13] Ohtani H, Yoshie O (2010). Morphometric analysis of the balance between CXCR3^+^ T cells and FOXP3^+^ regulatory T cells in lymphocyte-rich and conventional gastric cancers. Virchows Arch.

[14] Mizoi T, Ohtani H, Miyazono K, Miyazawa M, Matsuno S, Nagura H (1993). Immunoelectron microscopic localization of transforming growth factor β1 and latent transforming growth factor β1 binding protein in human cancer cells and stromal cells. Cancer Res.

[15] Kinugasa S, Abe S, Tachibana M (1998). Overexpression of transforming growth factor-beta1 in scirrhous carcinoma of the stomach correlates with decreased survival. Oncology.

[16] Vagenas K, Spyropoulos C, Gavala V, Tsamandas AC (2007). TGFbeta1, TGFbeta2, and TGFbeta3 protein expression in gastric carcinomas: correlation with prognostics factors and patient survival. J Surg Res.

[17] Sobin LH, Gospodarowicz MK, Wittekind CH (2009). UICC TNM classification of malignant tumours.

[18] Miyazono K, Olofsson A, Colosetti P, Heldin CH (1991). A role of the latent TGF-beta 1-binding protein in the assembly and secretion of TGF-beta 1. EMBO J.

[19] Chen W, Jin W, Hardegen N, Lei KJ, Li L, Marinos N, McGrady G, Wahl SM (2003). Conversion of peripheral CD4+CD25-naive T cells to CD4+CD25+ regulatory T cells by TGF-beta induction of transcription factor Foxp3. J Exp Med.

[20] Yang X, Letterio JJ, Lechleider RJ, Chen L, Hayman R, Gu H, Roberts AB, Deng C (1999). Targeted disruption of SMAD3 results in impaired mucosal immunity and diminished T cell responsiveness to TGF-beta. EMBO J.

[21] Kobayashi A, Weinberg V, Darragh T, Smith-McCune K (2008). Evolving immunosuppressive microenvironment during human cervical carcinogenesis. Mucosal Immunol.

[22] Bonertz A, Weitz J, Pietsch DH (2009). Antigen-specific Tregs control T cell responses against a limited repertoire of tumor antigens in patients with colorectal carcinoma. J Clin Invest.

[23] Dumitriu IE, Dunbar DR, Howie SE, Sethi T, Gregory CD (2009). Human dendritic cells produce TGF-beta 1 under the influence of lung carcinoma cells and prime the differentiation of CD4+CD25+Foxp3+ regulatory T cells. J Immunol.

[24] Guilliams M, Ginhoux F, Jakubzick C, Naik SH, Onai N, Schraml BU, Segura E, Tussiwand R, Yona S (2014). Dendritic cells, monocytes and macrophages: a unified nomenclature based on ontogeny. Nat Rev Immunol.

[25] Dieu-Nosjean MC, Goc J, Giraldo NA, Sautès-Fridman C, Fridman WH (2014). Tertiary lymphoid structures in cancer and beyond. Trends Immunol.

[26] Terabe M, Robertson FC, Clark K, de Ravin E, Bloom A, Venzon DJ, Kato S, Mirza A, Berzofsky JA (2017). Blockade of only TGF-β 1 and 2 is sufficient to enhance the efficacy of vaccine and PD-1 checkpoint blockade immunotherapy. Oncoimmunology.

[27] Ravi R, Noonan KA, Pham V, Bedi R, Zhavoronkov A, Ozerov IV, Makarev E, V. Artemov A, Wysocki PT, Mehra R, Nimmagadda S, Marchionni L, Sidransky D, Borrello IM, Izumchenko E, Bedi A (2018). Bifunctional immune checkpoint-targeted antibody-ligand traps that simultaneously disable TGFβ enhance the efficacy of cancer immunotherapy. Nat Commun.

