# Supplementary material for: Immune cell expression of TGFβ1 in cancer with lymphoid stroma: dendritic cell and regulatory T cell contact
Source: Virchows Arch. 2018 Mar 28;472(6):1021–8. doi: 10.1007/s00428-018-2336-y (PMC5999139; doi:10.1007/s00428-018-2336-y)
Supplement: Supplementary file 1 — Supplementary data in the Materials and Methods section. (DOCX 788 kb) [file 428_2018_2336_MOESM1_ESM.docx]

Appendix 1. Supplementary data in the Materials and Methods section

Fig. 5. Histopathological details of Ly-rich GCs.

Fig. 5-1. Typical LELC.


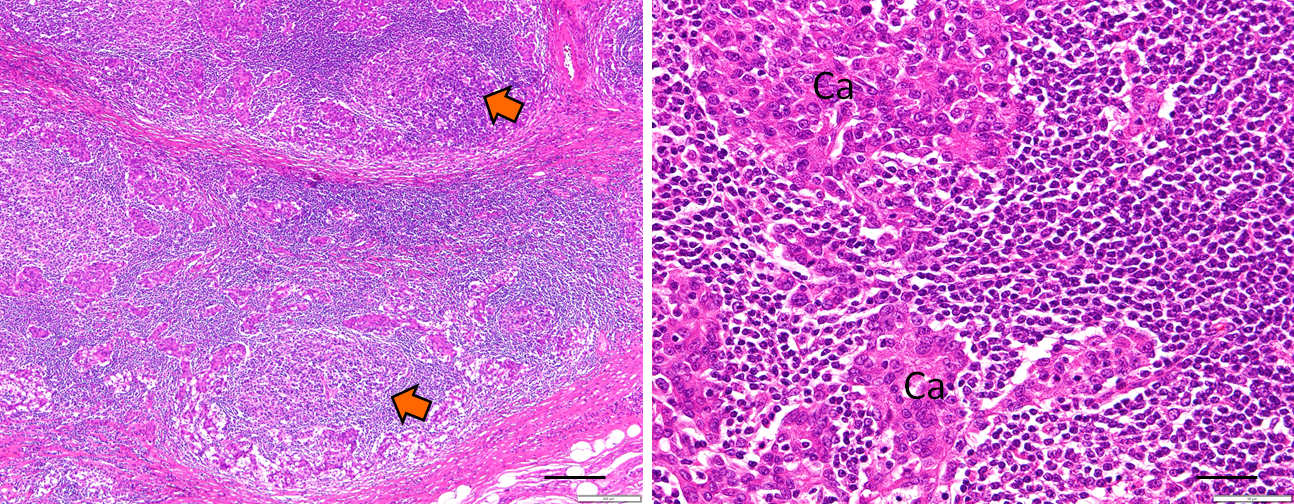


Both figures show prominent lymphoid stroma without deposition of collagen bundles. Lymphoid follicles with germinal center are formed (*Left*. red arrows). Ca (*right*), carcinoma cells. This case corresponds to “gastric cancer with lymphoid stroma” by Japanese classification. Scale bars, 200μm (*left*) and 50μm (*right*).

Fig. 5-2. LELC-like carcinoma


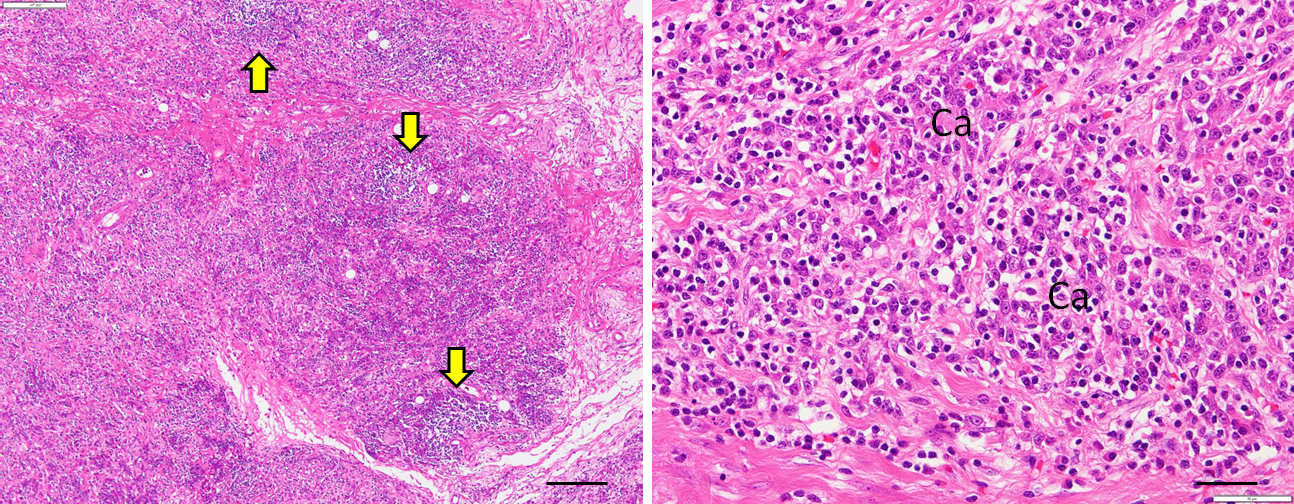


Both figures show that lymphocytes are distributed throughout the stroma less densely than in typical LELC. Note pushing margin (*left*) and aggregation of lymphocytes (*left* yellow arrows). Higher magnification (*right*) shows intraepithelial lymphocytes, which are, however, difficult to determine in immunohistochemical specimens. Therefore, we dealt mainly with stromal lymphocytes. This Ly-rich GC belongs to LELC-like cancer in this article. This case could be classified as “poorly differentiated adenocarcinoma (solid type)” by Japanese classification. Scale bars, 200μm (*left*) and 50μm (*right*). Ca, carcinoma cells.

**Histopathological details of Ly-rich GCs.**

Ly-rich GCs in this article include both lymphoepithelioma-like carcinoma [LELC] (synonym: gastric carcinoma with lymphoid stroma) and LELC-like cancer. Typical LELCs are characterized by 1) prominent lymphocyte-rich stroma usually with lymphoid follicles, 2) pushing margin, and 3) poorly-differentiated solid cancer cells. LELC-like cancer in this article means gastric cancer (GC) of any histological type infiltrated by lymphocytes in the whole stroma but insufficient for the criteria of LELC (the lymphoid stroma in this category may be less populated with lymphocytes than in LELC). Accumulation of lymphocytes along the invasive margin alone is not sufficient for its diagnosis. Most cases of typical LELC are positive for Epstein-Barr virus (EBV). But it is also important that EBV^+^ GCs do not always show lymphoid stroma.

Fig. 6. Immunohistochemistry for DNA mismatch repair proteins.


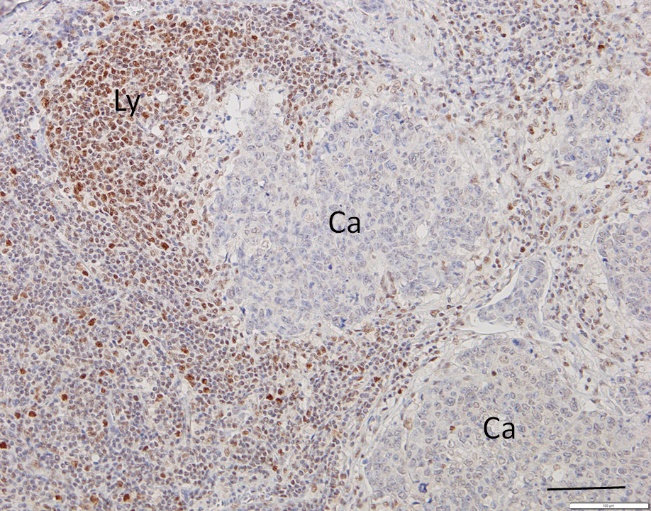


Negative result of MLH1 staining in cancer cells (Ca) is shown above in a typical case of LELC. Of nine cases of EBV^-^ Ly-rich GCs, three cases showed no HML1 expression in cancer cells (Ca) as contrasted by stromal lymphocytes (Ly) (suggesting microsatellite instability). Four cases were positive for MLH1, MSH6 and PMS2, not supporting microsatellite instability. Other two cases showed no reactivity (no judgement). Scale bar, 100μm. (Primary antibodies from BioGenex, Fremont, CA)

Table 1. Stage distribution of all cancer samples

| Ly-rich GCs (total) | |  |  |  |  |
| --- | --- | --- | --- | --- | --- |
| stage | 1 | 2 | 3 | 4 | total |
| numbers | 11 | 9 | 2 | 1 | 23 |
| (%) | 49% | 39% | 1% | <1 |  |
|  |  |  |  |  |  |
| Typical LELC (of Ly-rich GCs) | | | |  |  |
| stage | 1 | 2 | 3 | 4 | total |
| numbers | 10 | 3 | 1 | 0 | 14 |
| (%) | 71% | 21% | 7% | 0% |  |
|  |  |  |  |  |  |
| Control GCs (sequentially sampled) | | | | |  |
| stage | 1 | 2 | 3 | 4 | total |
| numbers | 10 | 13 | 8 | 4 | 35 |
| (%) | 29% | 37% | 23% | 11% |  |

The stage distribution of Ly-rich GCs tends to be more concentrated to stages 1 and 2, but not statistically significant. The stage distribution of typical LELC was concentrated to stage 1, as compared with that of control GCs (P<0.039, chi square method)

Table 2. Histological typing of control GCs (sequentially sampled).

| Histological types of control GCs | | | | |  |  |  |
| --- | --- | --- | --- | --- | --- | --- | --- |
| histology | tub | pap | por1 | por2 | sig | muc | total |
| numbers | 16 | 5 | 7 | 5 | 1 | 1 | 35 |

Abbreviations used: tub, tubular adenocarcinoma; pap, papillary adenocarcinoma; por1 and 2, poorly differentiated adenocarcinoma of solid- and non-solid types, respectively; sig, signet ring cell carcinoma; muc, mucinous adenocarcinoma.

Fig. 7. Follow-up analysis

Disease-specific survival of 23 Ly-rich GCs in Mito Medical Center


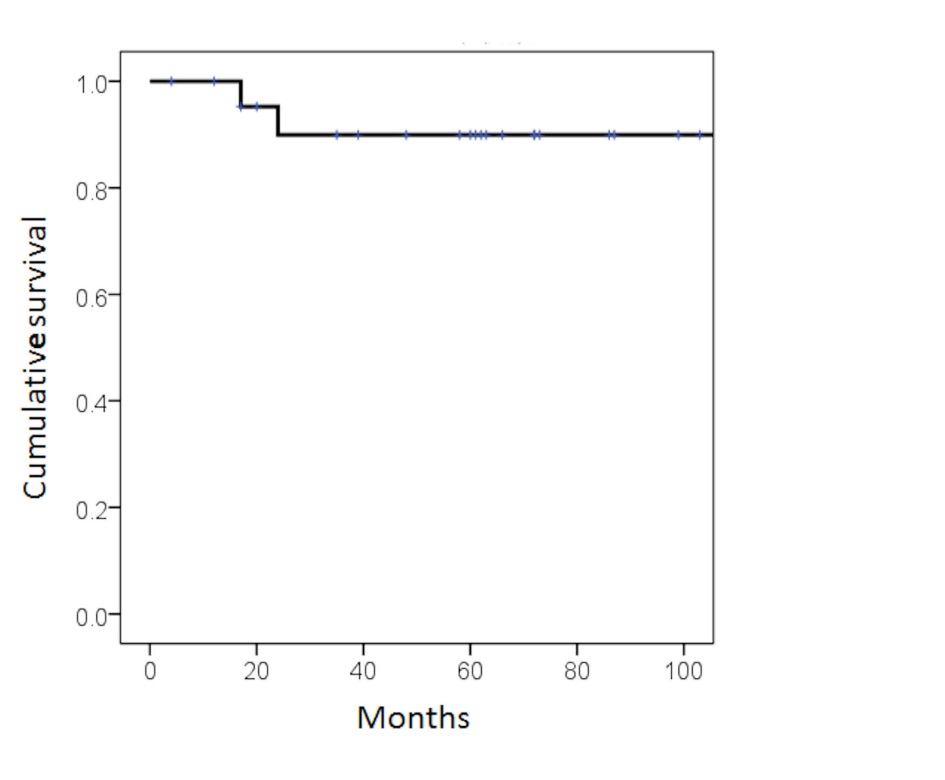


Mean survival, 103.2 months (95% CI, 82.8-123.7 months).

(Survival analysis by SPSS version 21)
