# Supplementary material for: Immune cell expression of TGFβ1 in cancer with lymphoid stroma: dendritic cell and regulatory T cell contact
Source: Virchows Arch. 2018 Mar 28;472(6):1021–8. doi: 10.1007/s00428-018-2336-y (PMC5999139; doi:10.1007/s00428-018-2336-y)
Supplement: Supplementary file 2 — Supplementary data in the Results section. (DOCX 4299 kb) [file 428_2018_2336_MOESM2_ESM.docx]

Appendix 2 (Figs. 8-10). Supplementary data in the Results section.

Fig. 8-1. Negative control for LAP (TGFβ1) immunohistochemistry in Ly-rich GC


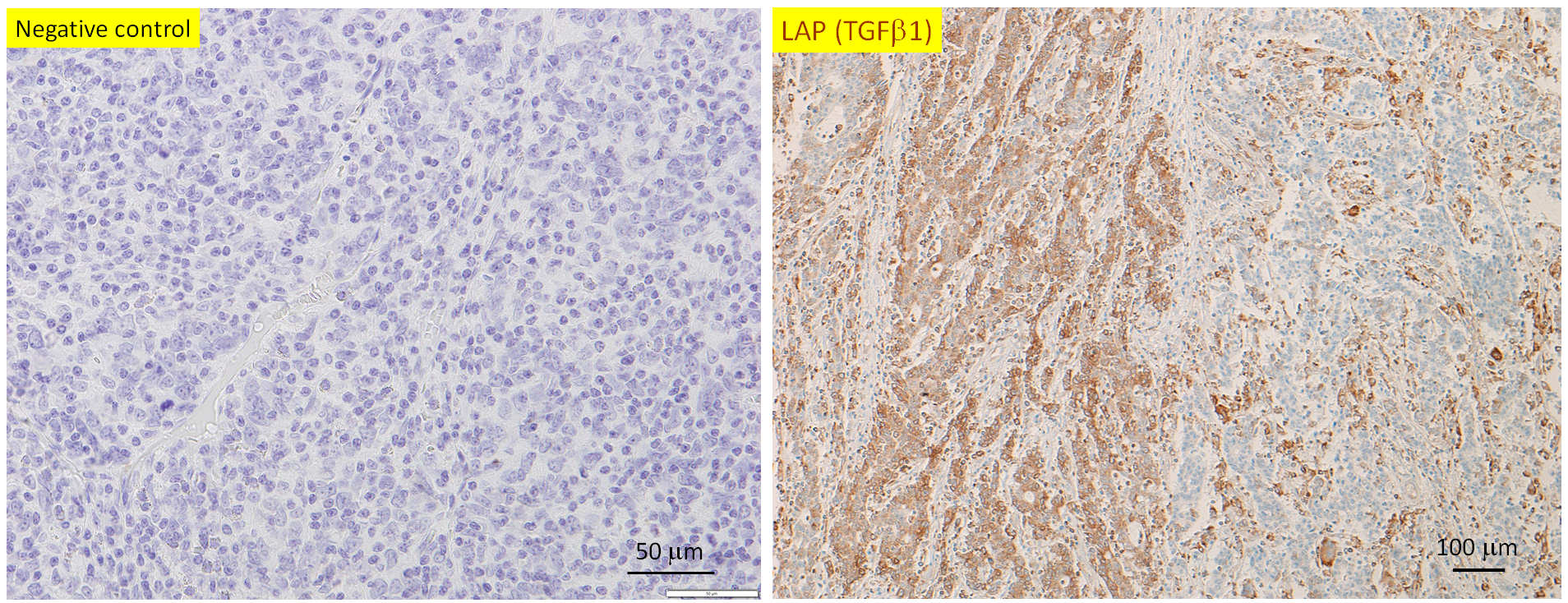


The primary antibody against LAP (TGFβ1) was replaced by the same concentration of non-immunized goat IgG (0.5μg/ml). No signals are observed.

Fig. 8-2. LAP (TGFβ1) immunohistochemistry in control GCs


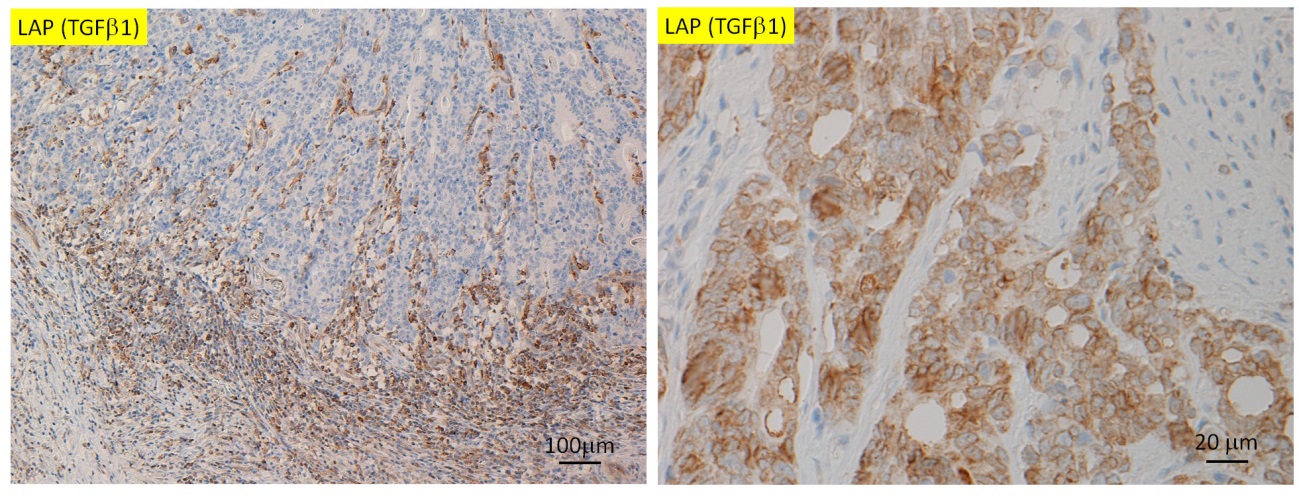


LAP (TGFβ1) is expressed in immune cells (*left*) along invasive margin (tumor-host interface) and in cancer cells (*right*) in control tubular adenocarcinoma.

Fig. 8-3. Identification of LAP (TGFβ1)^+^ cells as macrophages and/or immature classical/conventional dendritic cells (cDCs) in Ly-rich GCs


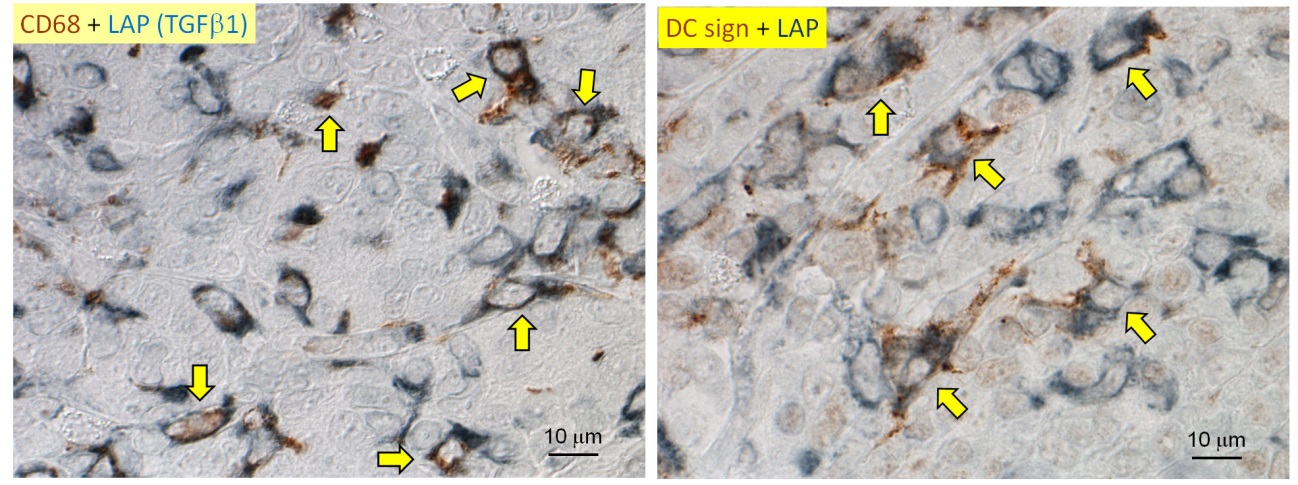


Arrows indicate double positive cells for CD68 (brown) and LAP (dark blue)(*left*) and DC sign (brown) and LAP (dark blue)(*right*) in Ly-rich GCs. (double-labeling chromogenic immunohistochemistry).

Fig. 8-4. Infrequent expression of LAP (TGFβ1) in FoxP3^+^ cells by double immunofluorescent microscopy.

Most of FoxP3^+^ cells (red) did not co-express LAP (TGFβ1) (green) in this Ly-rich GC. Merged figure by conventional fluorescent microscopy. No DAPI nuclear staining.

Fig. 8-5. LAP (TGFβ1) expression in the secondary lymphoid organs (A)


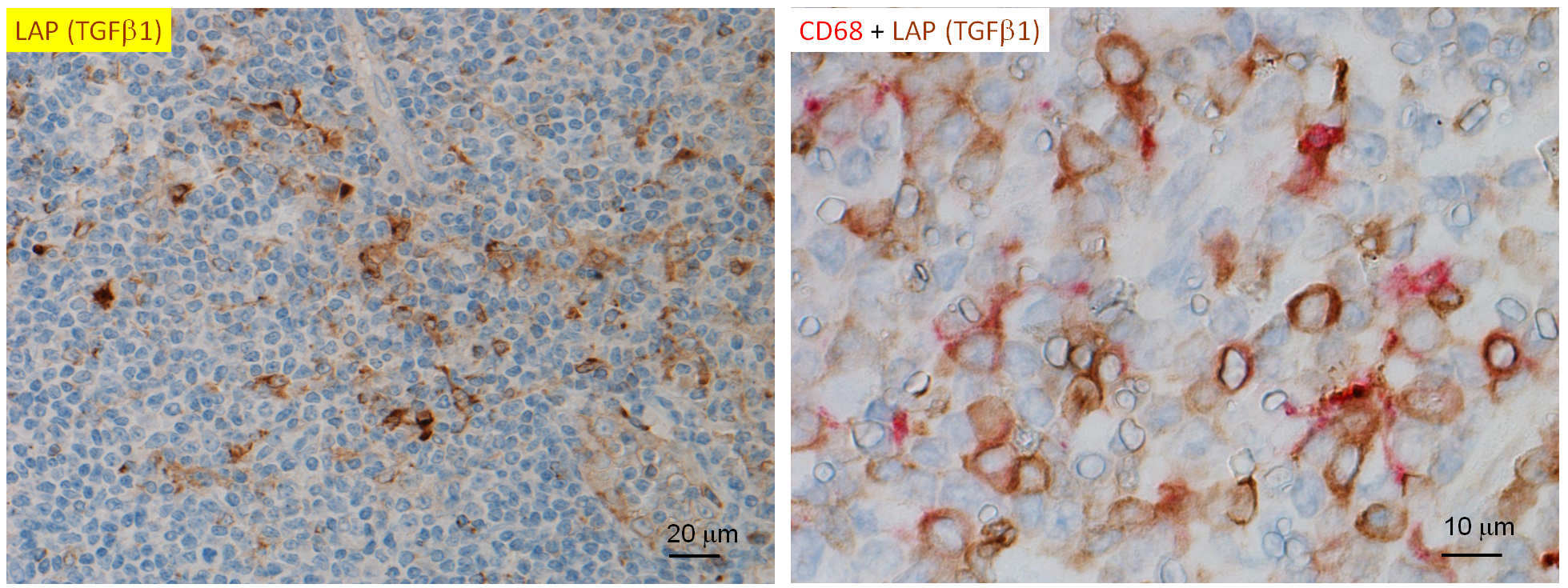


*Left,* LAP (TGFβ1) is expressed in dendritic-shaped, oval or round cells in the T-cell zone (paracortex) of a mesenteric lymph node. *Right,* double chromogenic staining for CD68 (red) and LAP (TGFβ1) (brown) shows that most of CD68^+^ macrophages (including immature cDCs) co-express LAP (TGFβ1) in the T-cell zone of tonsil.

Fig. 8-6. LAP (TGFβ1) expression in the secondary lymphoid organs (B)


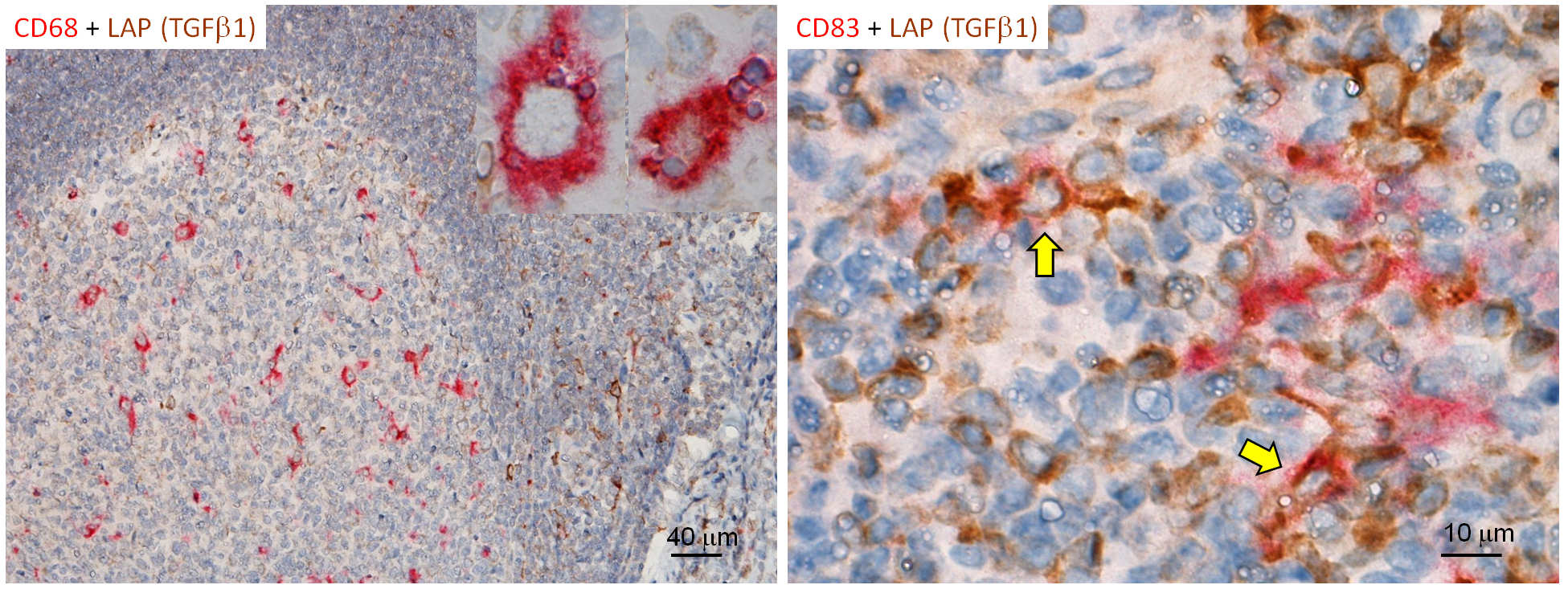


*Left*, LAP (TGFβ1) is NOT expressed in the CD68^+^ macrophages (red) in the germinal center of the appendix, which are clearly shown in the inset at a higher magnification. This finding was common in every secondary lymphoid organs observed and also in cancer stroma. *Right*, double staining for CD83 (red) and LAP (TGFβ1) (brown) in the T-cell zone of the tonsil. Note the presence of double positive cells (arrows), which indicate TGFβ1^+^ mature cDCs.

Fig. 8-7. Relationship between LAP^+^ dendritic-shaped cell and T_reg_ cells in Ly-rich GCs.


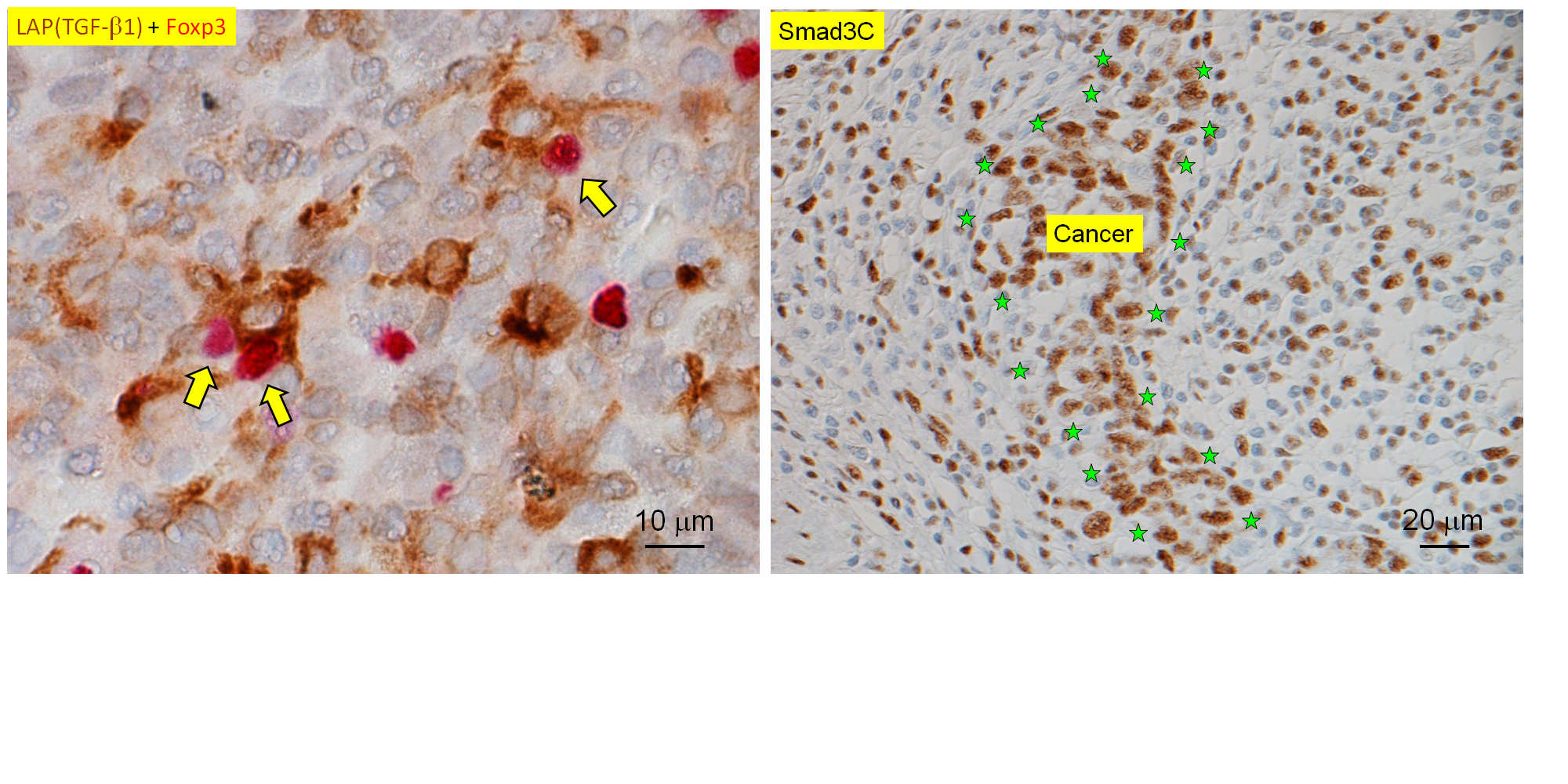


LAP (TGFβ1)^+^ dendritic-shaped cells (brown) harbor FoxP3^+^ T_reg_ cells (red) in a close cell-to-cell contact (arrows) in Ly-rich GC by double chromogenic immunohistochemistry.

Fig. 8-8. Immunohistochemistry for Smad3C and negative control in Ly-rich GCs


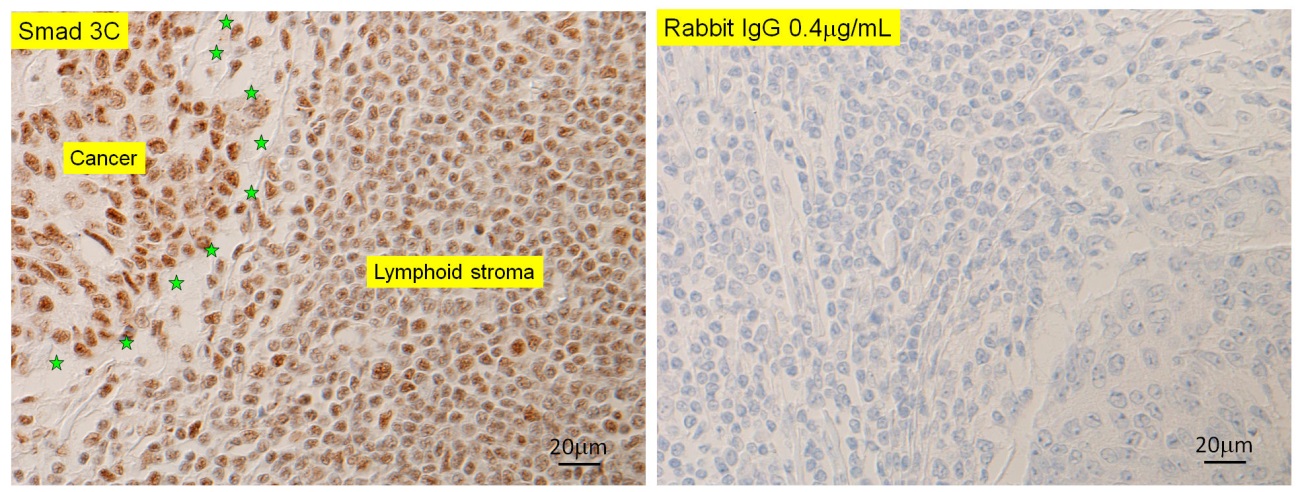


*Left*, Smad3C is widely expressed in both cancer cells and lymphocytes in Ly-rich GCs. *Right*, negative control shows no signals.

Fig. 9. Statistical analyses on LAP (TGFβ1) positivity in classical/conventional dendritic cells (cDCs) by double chromogenic immunohistochemistry.

A B


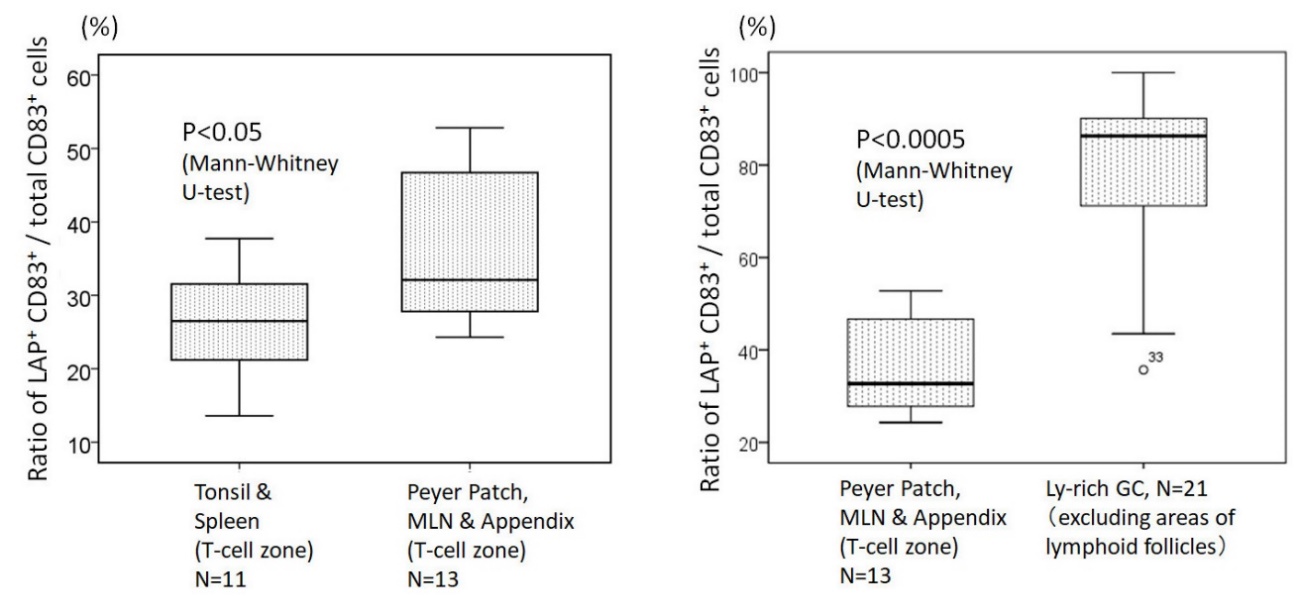


C


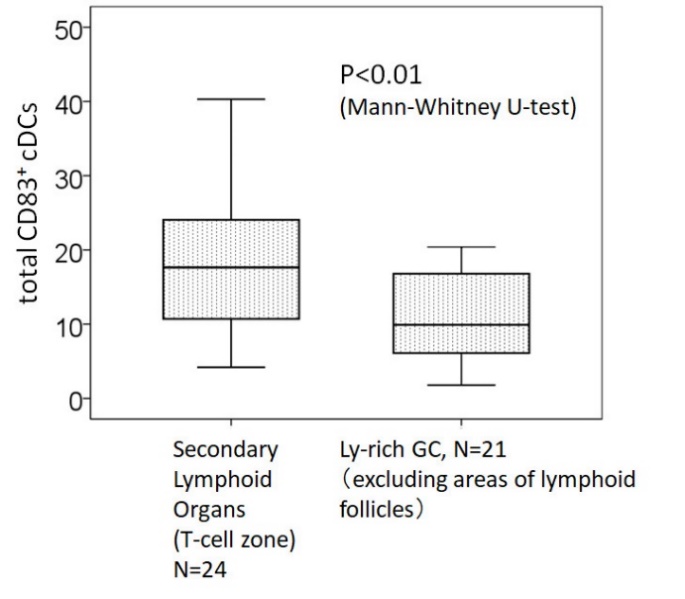


Fig. 9a. Among the secondary lymphoid organs, the ratio of LAP (TGFβ1)^+^ CD83^+^ cDCs per total CD83^+^ cDCs is higher in the T cell zone of the gut-associated lymphoid organs (Peyer patches, mesenteric lymph nodes [MLN] and appendix vermiformis) than in the T cell zone of the tonsil and spleen.

Fig. 9b. The ratio of LAP (TGFβ1)^+^ CD83^+^ cDCs per total CD83^+^ cDCs in Ly-rich GCs is still higher than in the T cell zone of gut-associated lymphoid organs (Peyer patches, MLN and appendix vermiformis).

Fig. 9c. The number of total CD83^+^ cDCs is higher in the T cell zone of secondary lymphoid organs (as a total) than in Ly-rich GCs

Fig. 10. Correlation analyses between lymphocytes and cDCs in Ly-rich GCs by single or double chromogenic immunohistochemistry.

A B


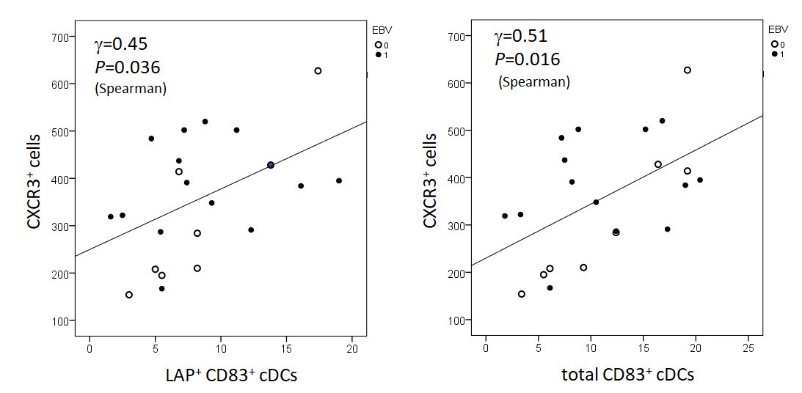


C D


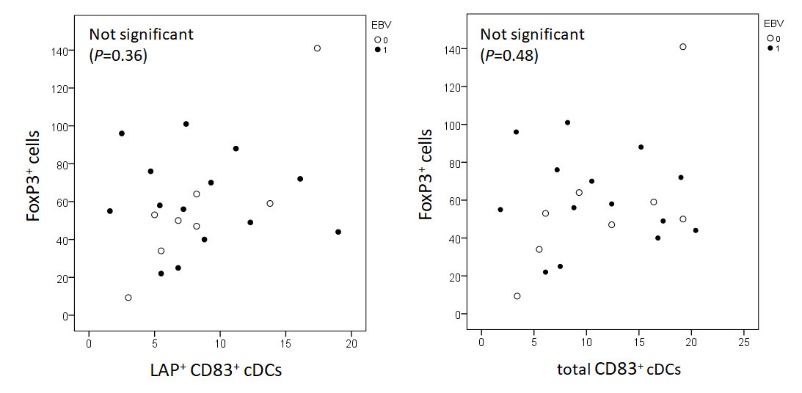


Fig. 10a, b (upper). The number of CXCR3^+^ cells positively correlates with that of LAP (TGFβ1)^+^ CD83^+^ cDCs (a) and that of total CD83+ cDCs (b) in Ly-rich GCs.

Fig. 10c, d (lower). The number of FoxP3^+^ cells does not correlate with that of LAP (TGFβ1)^+^ CD83^+^ cDCs (c) and that of total CD83^+^ cDCs (d) in Ly-rich GCs.

Black circles, EBV^+^ Ly-rich GCs. Open circles, EBV^-^ GCs.

The number is expressed as number per each unit area in this study (0.0625 mm^2^).
